# Supplementary material for: Individual uniqueness of connectivity gradients is driven by the complexity of the embedded networks and their dispersion
Source: Brain Struct Funct. 2025 Jul 3;230(6):110. doi: 10.1007/s00429-025-02976-8 (PMC12226633; doi:10.1007/s00429-025-02976-8)
Supplement: Supplementary file 1 — Supplementary Material 1 [file 429_2025_2976_MOESM1_ESM.docx]

# **Supplementary Materials**


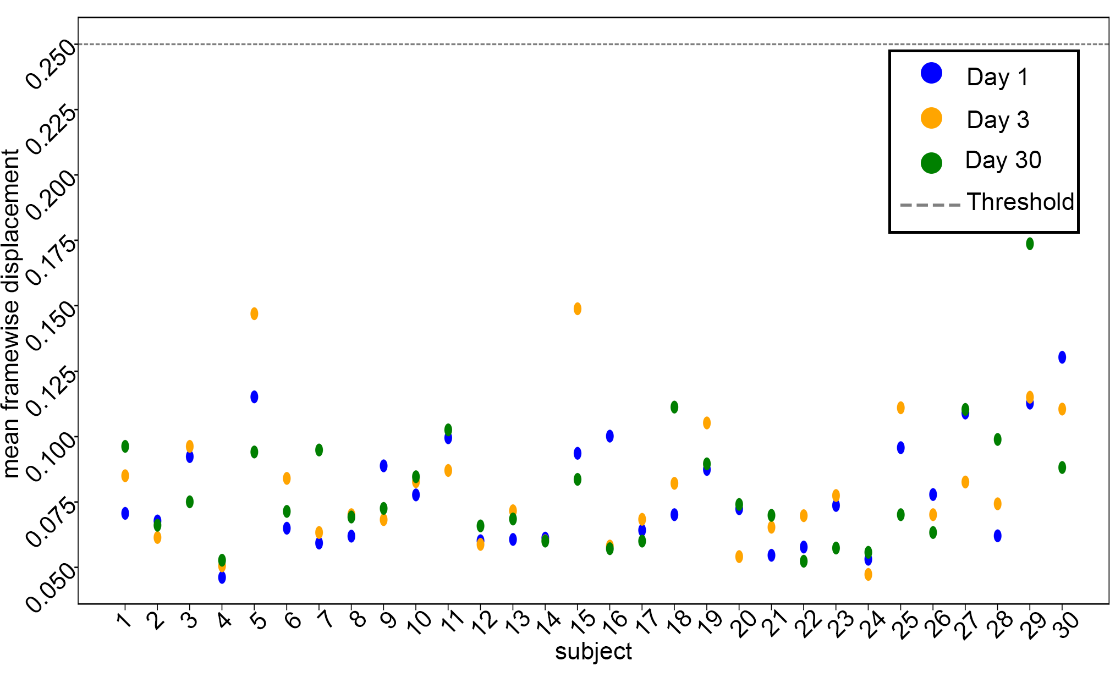


***Fig S1. Mean framewise displacement (FD) across scan days for each participant.*** Each dot represents the mean FD value for a single subject on Day1 (blue), Day3 (orange), and Day30 (green). The dashed horizontal line marks the exclusion threshold of 0.25 mm. All participants remained below this threshold in all sessions.


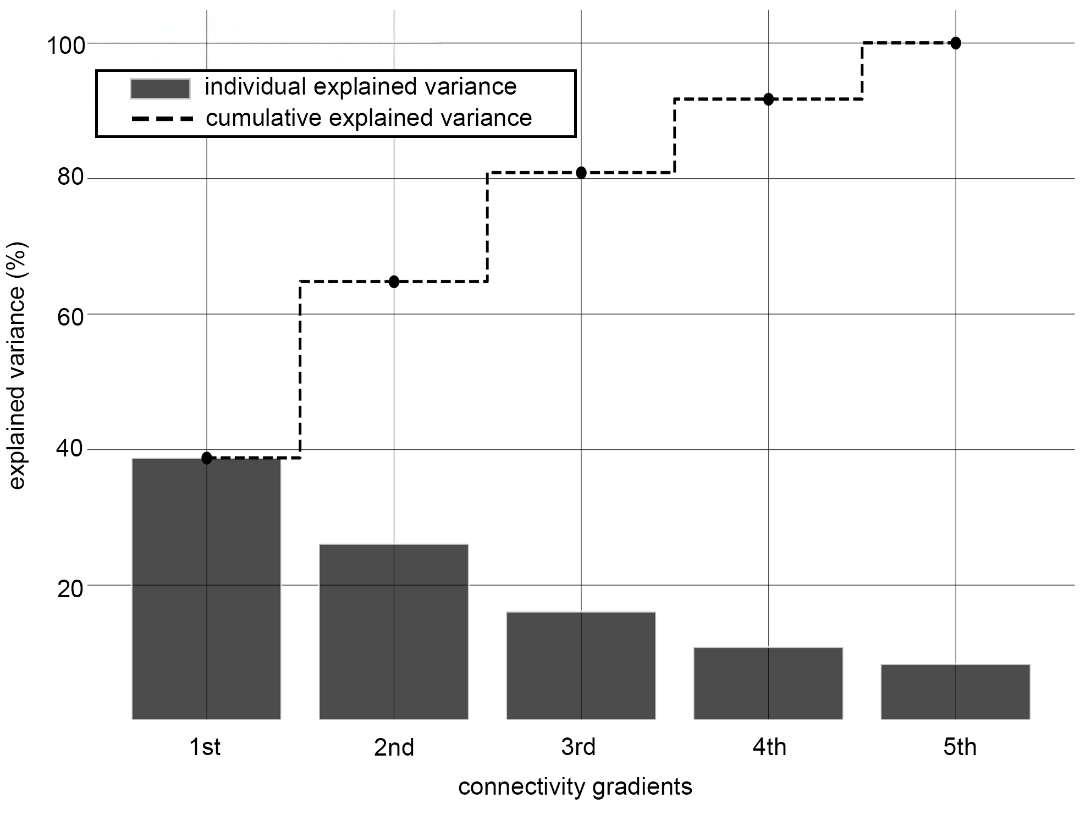


***Fig. S2 Individual and cumulative explained variance by connectivity gradients****.* Percent explained variance for each component out of the 5 components decomposed in our analysis. The dashed line indicates the cumulative explained variance. The principal component accounts for the largest proportion of variance, at 38%. Notably, the cumulative explained variance approaches 81% by the third component.

**Supplementary material M1. Deriving connectivity gradients based on diffusion embedding (DE)**

To investigate if our results are generalized to an additional, commonly used dimensionality technique (Hong et al., 2020), the full analysis pipeline was performed using diffusion embedding as implemented in the default settings of BrainSpace (Vos de Wael et al., 2020). This included a sparsity threshold of 0.9 to retain the 10% of the strongest connections. Importantly, all main results were replicated (Fig. S3 – Fig. S7) thereby supporting robustness of the findings irrespective of the algorithm used for dimensionality reduction.


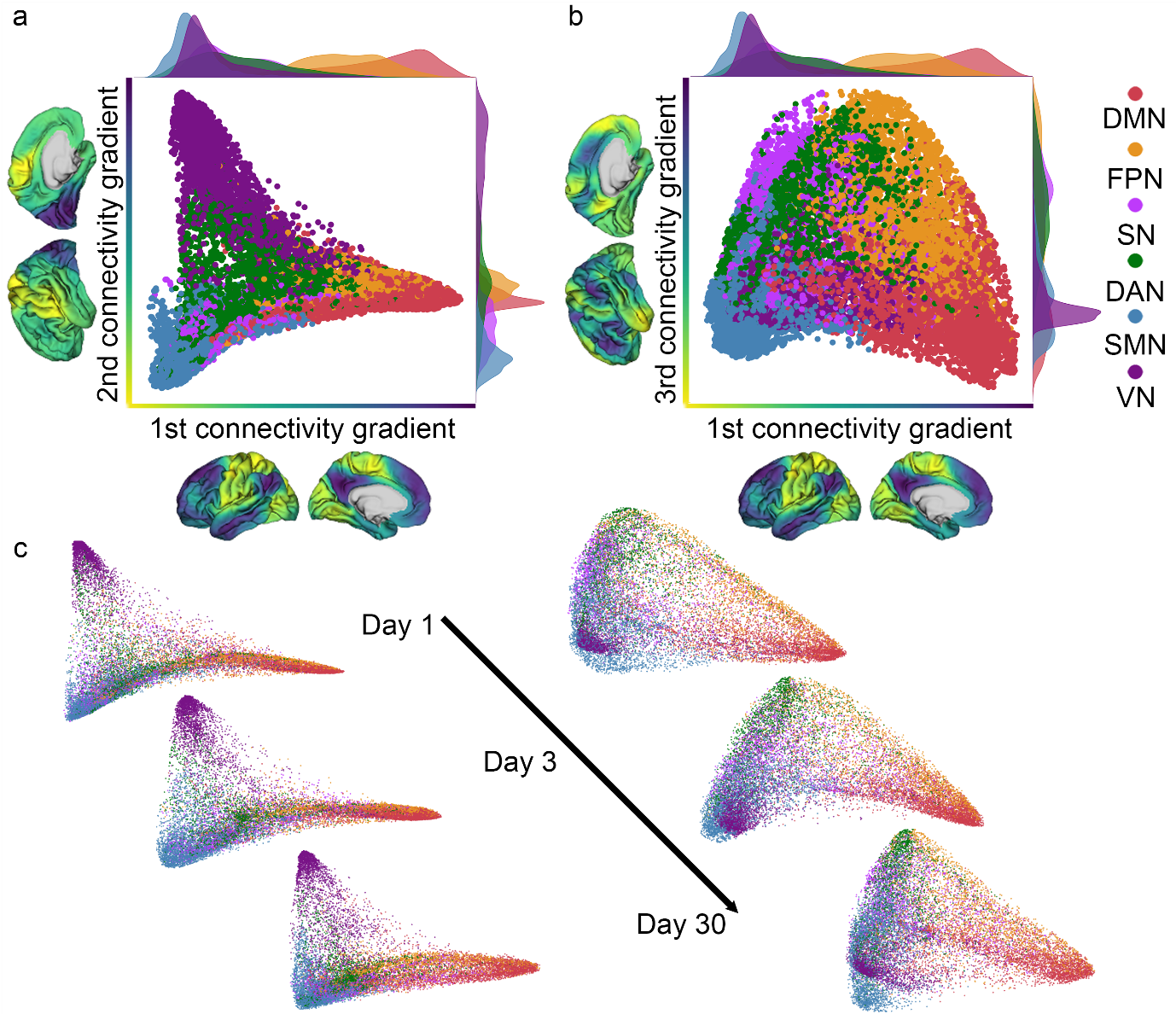


***Fig S3. Connectivity gradients derived using diffusion embedding (DE) replicate the spatial organization observed with PCA.*** (a) Group-level gradient map along the first and second connectivity gradients, with vertices colored by Yeo’s 7-network parcellation (Thomas Yeo et al., 2011). The first gradient (x-axis) captures a hierarchical organization from the default-mode network (DMN) to sensorimotor (SMN) and visual (VN) networks, while the second gradient (y-axis) separates the VN from other networks. (b) Gradient map along the first and third gradients. The third gradient (y-axis) differentiates fronto-parietal (FPN), dorsal attention (DAN), and salience (SN) networks from the DMN. (c) Individual-level connectivity gradients for a representative subject across three timepoints (Days 1, 3, and 30), showing stable yet subtly varying structures over time. These results confirm that the spatial patterns observed using PCA (Figure. 1 in main text) are similar to those obtained with diffusion embedding.


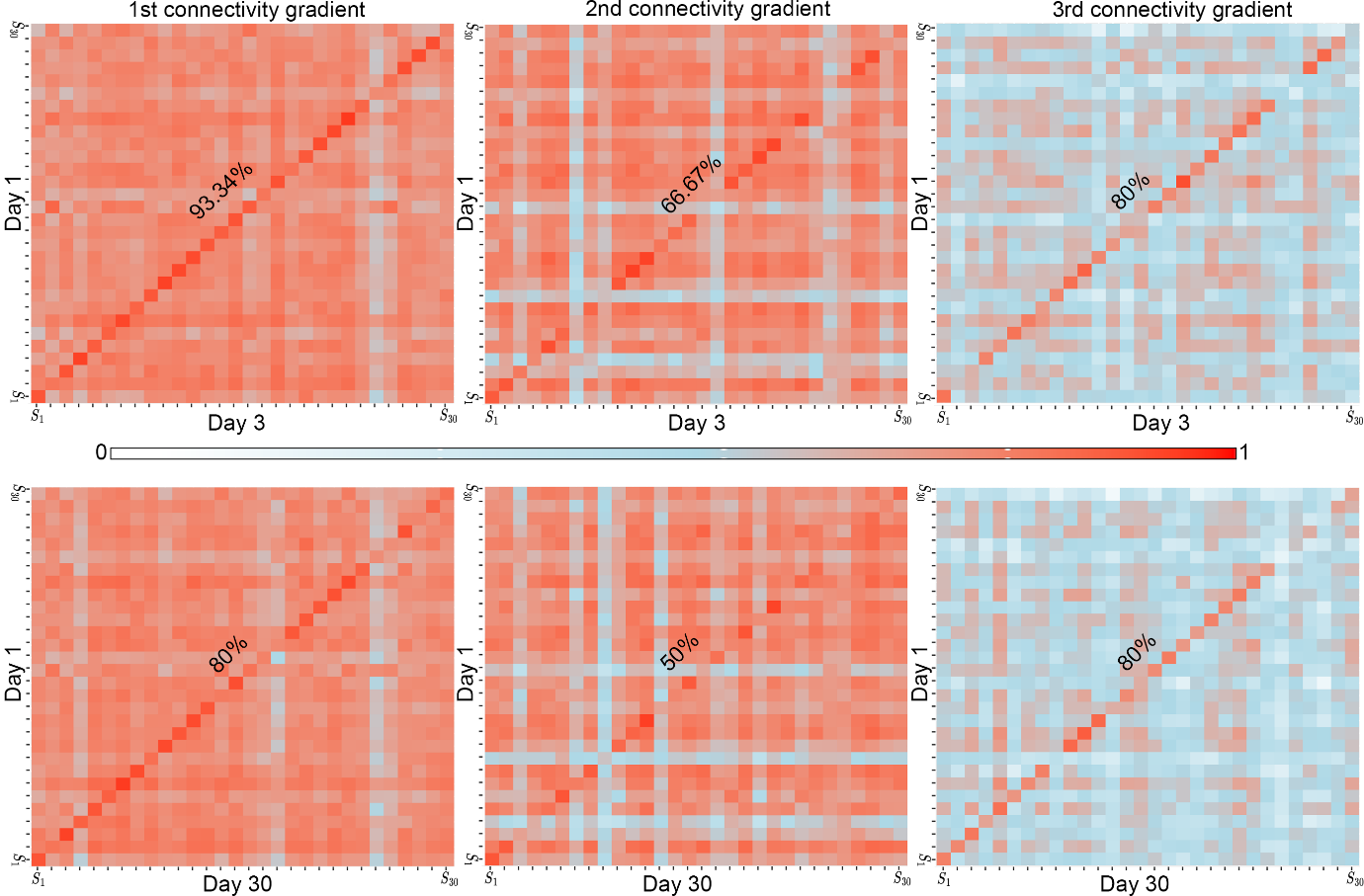


***Fig S4. Connectivity gradients computed using diffusion embedding (DE) show high within-subject stability over time.*** Pearson’s correlation coefficient pairwise values are shown for each of the first three gradients, comparing individual-level maps between sessions (day 1 with day 3, top row; day 1 with day 30, bottom row). High values along the diagonal indicate strong within-subject consistency. Identification analysis based on DE-derived gradients revealed high accuracy for Gradient 1 (93.34% for Day 1–3; 80% for Day 1–30), moderate accuracy for Gradient 2 (66.67% for Day 1–3; 50% for Day 1–30), and stable performance for Gradient 3 (80% for both intervals). These results replicate the results observed using PCA (see Fig. 2 in the main text), confirming that the individual-specific topography of connectivity gradients is robust to the choice of embedding method.


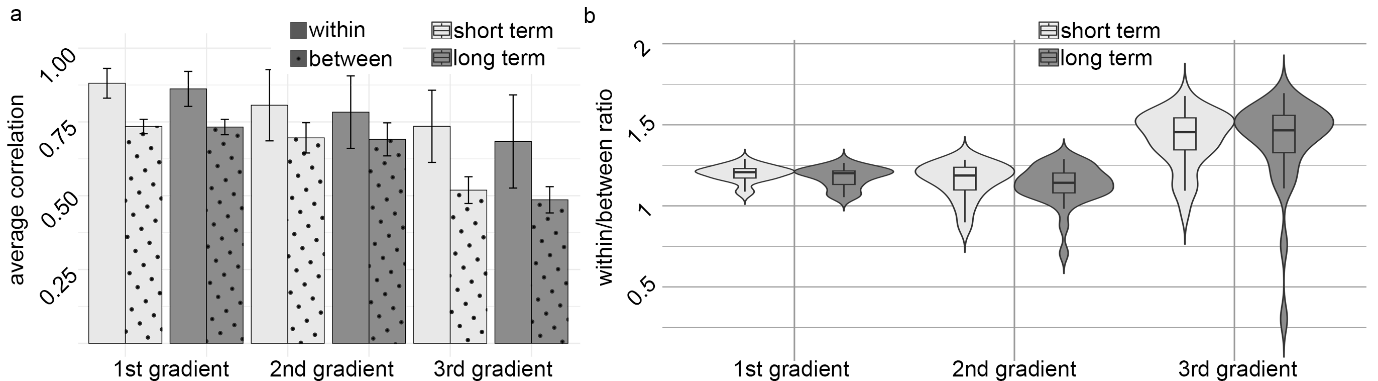


***Fig S5. Diffusion embedding (DE) reveals similar pattern of within/between correlation ratio to those found using PCA.*** (a) Mean Pearson correlations for short- and long-term intervals across gradients, comparing within- and between-subject similarity. (b) Violin plots show within-to-between correlation ratios, with values >1 indicating individual specificity. A significant main effect of gradient was found (F(2,177) = 60.44, $p_{BON}$ = 2e-16). Post-hoc tests showed that the third gradient differed significantly from the first and second (p < .0001), replicating a similar pattern to the one observed when using PCA (Fig. 3).


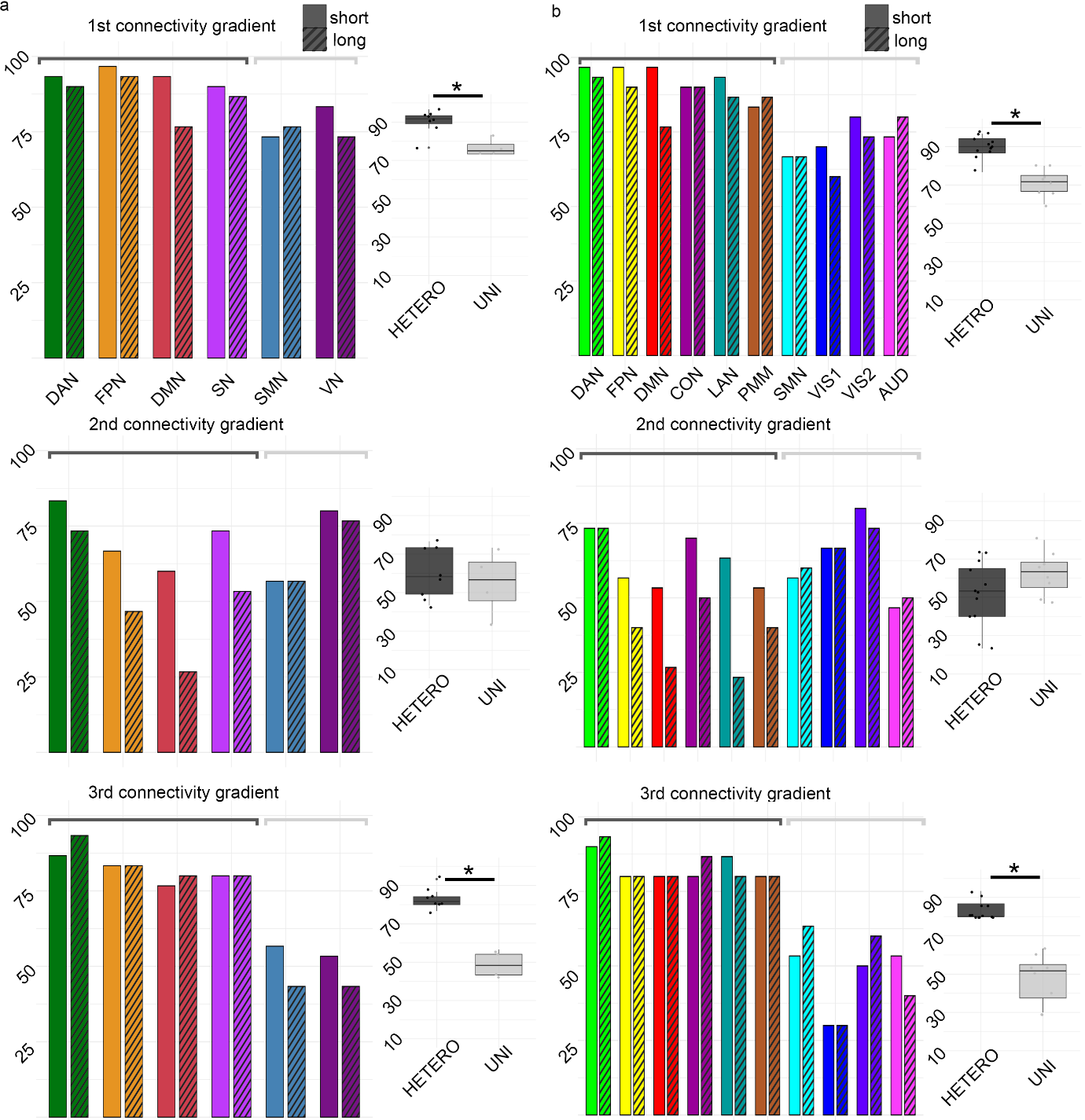


***Fig S6. Diffusion embedding (DE) reveals higher individual identification accuracy in heteromodal networks.*** Identification accuracy is shown for short-term (striped bars) and long-term (solid bars) sessions across functional networks for each of the first three connectivity gradients. Box plots (right) compare accuracy between heteromodal (HETERO) and unimodal (UNI) networks. (a) Yeo parcellation: Dorsal Attention Network (DAN), Frontoparietal Network (FPN), Default Mode Network (DMN), Salience Network (SN), Somatomotor Network (SMN), and Visual Network (VN). (b) Cole parcellation: DAN, FPN, DMN, Cingulo-Opercular Network (CON), Language Network (LAN), Posterior Multimodal Network (PMM), SMN, Visual Network 1 (VIS1), Visual Network 2 (VIS2), and Auditory Network (AUD). Heteromodal networks consistently showed higher identification accuracy than unimodal networks along the first and third gradients, with statistically significant differences after Bonferroni correction for multiple comparisons ($p_{BON}$ < 0.05). No significant difference was found for the second gradient. These findings replicate the general trends observed with PCA (Fig. 4), confirming the robustness of the relationship between network complexity and individual specificity across embedding methods.


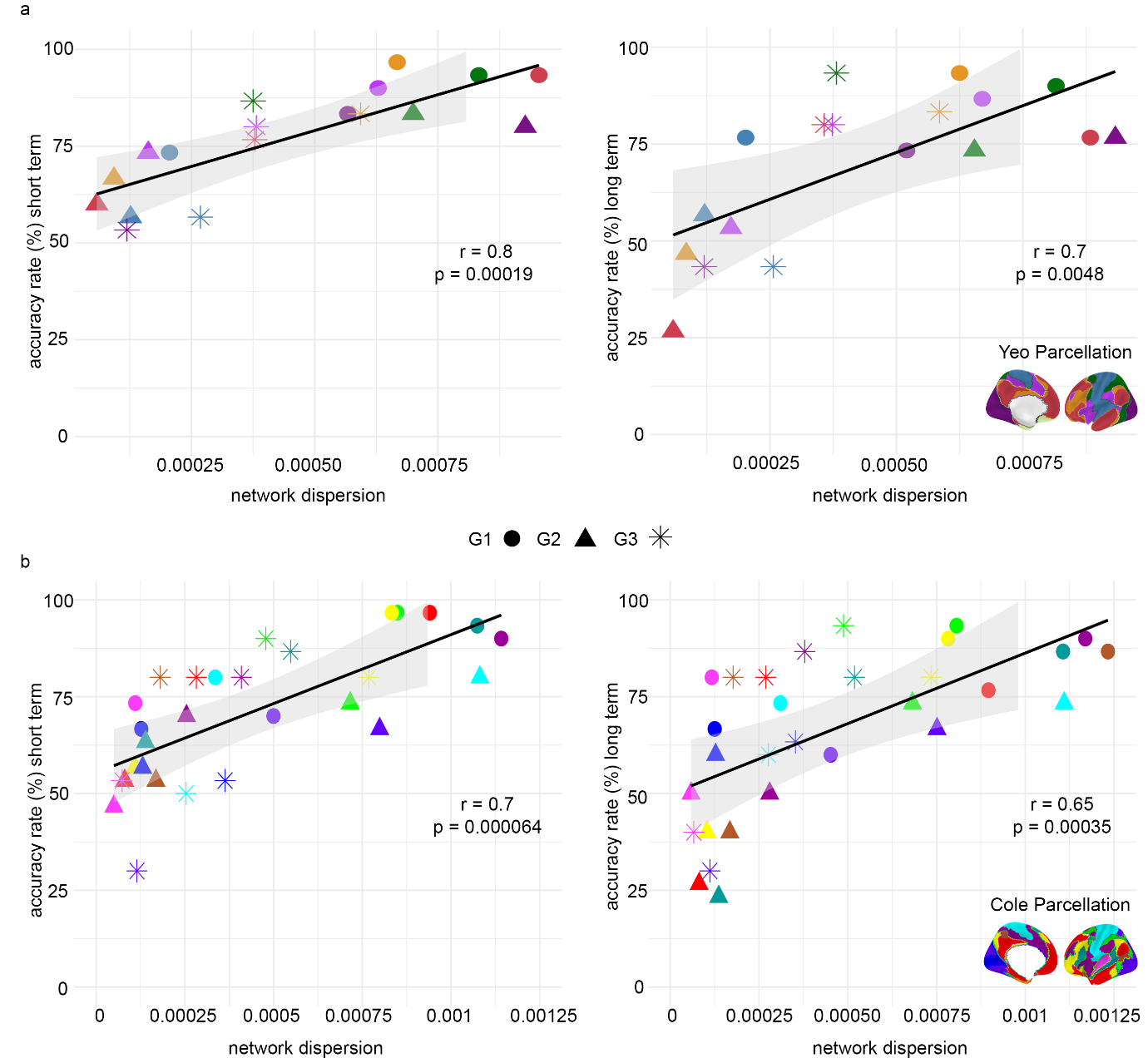


**Fig S7.** ***Identity accuracies in networks positively correlate with their dispersion along gradients derived via diffusion embedding.*** Each point represents a different network, with associated identification accuracy on the y-axis and network dispersion on the x-axis. Results are presented separately for short-term (Day1–Day3; left panels) and long-term (Day1–Day30; right panels). Dispersion values reflect the variance in gradient scores computed for each network parcel averaged for Day1 and Day3 or Day1 and Day30, respectively. (a) Yeo parcellation (Thomas Yeo et al., 2011); (b) Cole parcellation (Ji et al., 2018). Significant positive correlations (Bonferroni-corrected) were observed across all conditions, showing a consistent association between network dispersion and identification accuracy within this alternative approach. Brain maps illustrate corresponding networks for each parcellation.

**Supplementary material M2. The influence of variation in preprocessing strategy and threshold applied to connectivity matrices**

To examine how our results were influenced by preprocessing decisions, the main analysis (pair-wise Pearson’s correlation and identification accuracy) was performed for data preprocessed with additional removal of global signal regression. Gradient-based identification matrices were computed for the first three gradients by correlating gradient maps between Day 1 and Day 3 (short-term, top row of Fig S8) and between Day 1 and Day 30 (long-term, bottom row of Fig S8). Identification accuracies remained consistent with the main results, with the 1st and 3rd gradients showing the highest stability across both intervals (Day 1–Day 3: 86.67% and 86.67%; Day 1–Day 30: 83.34% and 80%). These findings suggest that the general pattern of results is similar with and without this preprocessing step.

To evaluate the influence of the affinity matrix sparsity threshold on identification accuracy, we performed the gradient-based identification analysis using a range of sparsity levels (0%, 50%, 90%, 95%, 99%). In our main analysis, the commonly used 90% threshold implemented in BrainSpace was used (Vos de Wael et al., 2020). This decision was driven by both the goal of reducing the risk of over-thresholding and a matching of preprocessing between current data and the template used for alignment. Identification accuracies remained relatively stable across thresholds, except for a reduction evident at 99% threshold (Fig. S9). Importantly, identification accuracy was high at both 90% and 95% threshold, with a slight increase at 95% for specific gradients. These results suggest that extremely sparse representations may hinder reliable gradient estimation.


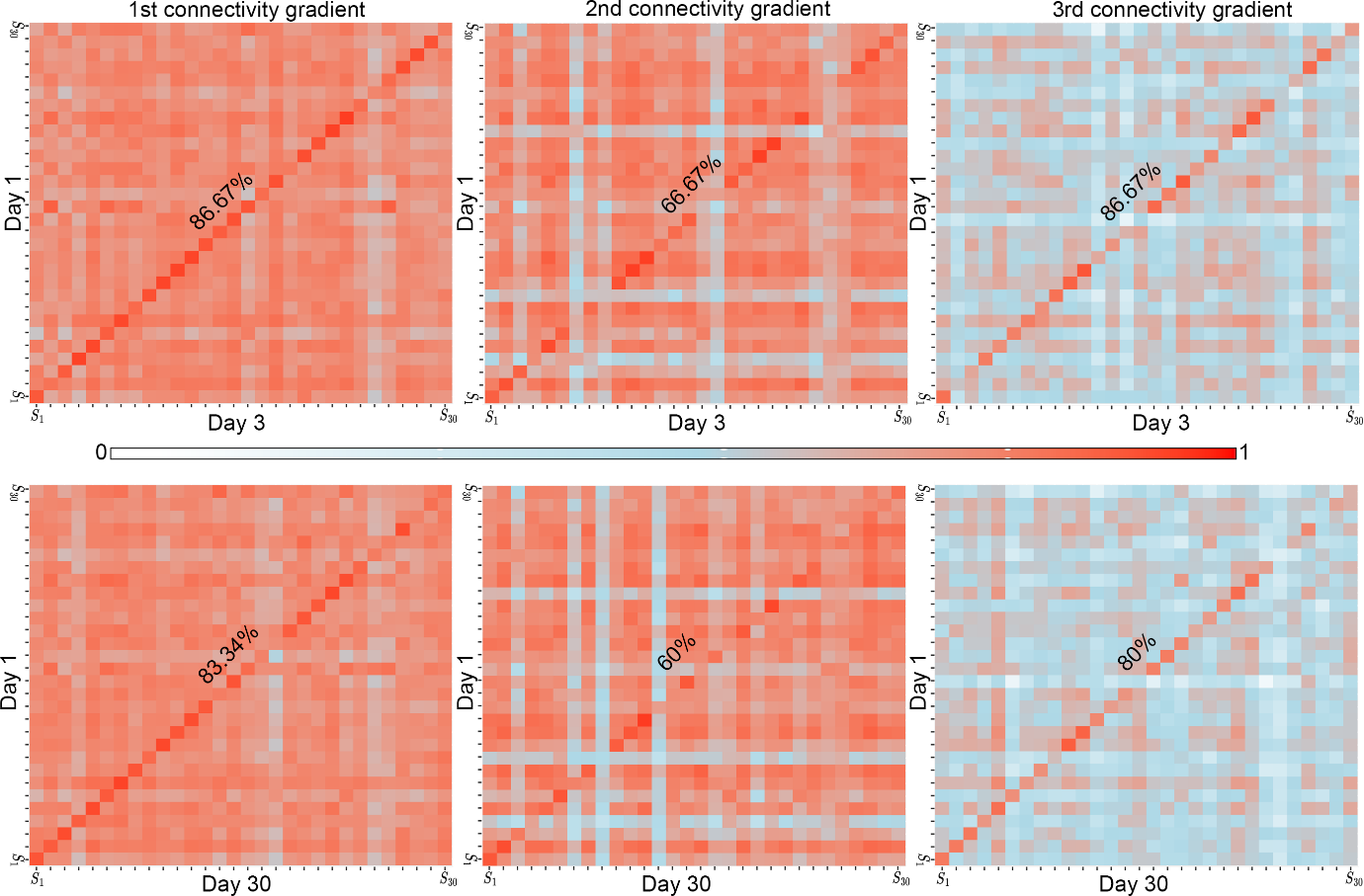


**Fig S8.** ***Identification accuracy matrices across short- and long-term intervals for the first three connectivity gradients using additional global signal regression during preprocessing.*** Pearson’s correlation coefficient pair-wise values between individual gradient maps across sessions, with higher diagonal values indicating higher within subjects’ similarity over time. Day 1–Day 3 correlations (short-term, top row), and Day 1–Day 30 correlations (long-term, bottom row). Identification accuracies remained consistent with the main results, with higher accuracies for the first and the third gradients and overall significant accuracies, as determined by a non-parametric permutation test (1,000 iterations). all resulting p-values were Bonferroni-corrected for multiple comparisons and remained significant (adjusted p = 0.006), suggesting this preprocessing step has no significant influence over the main results.


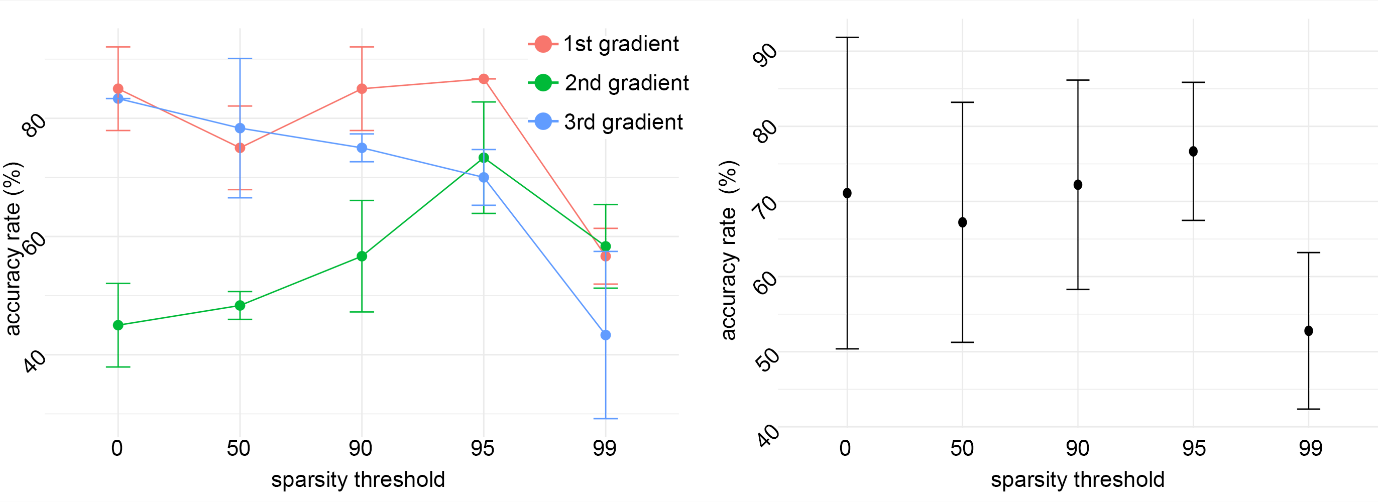


***Fig S9. Identification accuracy as a function of affinity matrix sparsity threshold.*** Identification accuracy rates for each of the first three connectivity gradients (G1–G3) across different sparsity thresholds (0%, 50%, 90%, 95%, 99%) applied to the affinity matrix prior to gradient decomposition, values represent the average of short- and long-term scores, and error bars indicate the standard deviation (STD) across these two time points. (left panel). Mean identification accuracy across gradients and time intervals with standard deviation. Identification accuracies remained relatively stable and high across thresholds, except for a reduction evident at 99% threshold. Importantly, identification accuracy was high at both 90% and 95% threshold, with a slight increase at 95% for specific gradients.

**Supplementary material M3. Identification rates based on three gradients’ combined spatial patterns**

To examine the potential utility of the usage of the joined information obtained over the first three gradient, gradient score values obtained via PCA decomposition were concatenated for each subject and each session. Pair-wise Pearson’s correlation for short- and long-time intervals was calculated, and ID accuracy scores was calculated as dome for the main analysis. The identification rates obtained (Fig. S10) in this manner were high and comparable to those obtained using the first gradient, and slightly higher for the combined information from all gradients (90% for the combined short-term interval and 90 % for the first gradient, and 83.34% for the combined long-term interval and 80 % for the first gradient). These results support the combined usage of gradient measures previously employed by others (Bethlehem et al., 2020) and further supports the contribution of the first connectivity gradients to the overall identification rate observed while taking all gradient information into account.


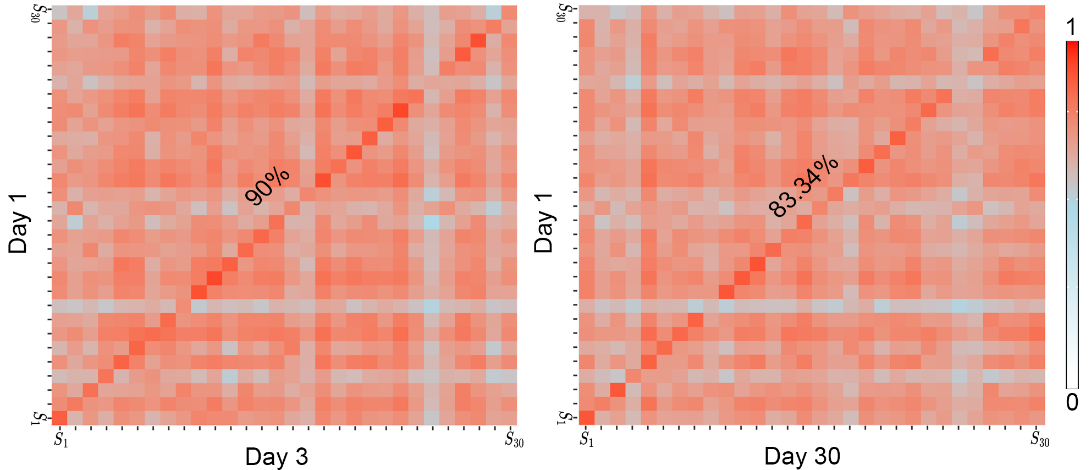


***Fig S10. Identification accuracy based on concatenated gradient features across short- and long-term intervals***. Each matrix displays Pearson correlation values between subjects' concatenated gradient vectors (combining the 1st, 2nd, and 3rd connectivity gradients) across sessions. The left panel shows correlations between Day 1 and Day 3 (short-term), while the right panel shows Day 1 to Day 30 (long-term). Identification accuracy, indicated on the diagonal, was 90% for the short-term and 83.34% for the long-term interval. These results suggest that combining multiple gradients may enhance individual specificity and support a slightly improved identification accuracy across time.

References:

Bethlehem, R. A. I., Paquola, C., Seidlitz, J., Ronan, L., Bernhardt, B., Consortium, C.-C., & Tsvetanov, K. A. (2020). Dispersion of functional gradients across the adult lifespan. *NeuroImage*, *222*, 117299. https://doi.org/10.1016/j.neuroimage.2020.117299

Hong, S. J., Xu, T., Nikolaidis, A., Smallwood, J., Margulies, D. S., Bernhardt, B., Vogelstein, J., & Milham, M. P. (2020). Toward a connectivity gradient-based framework for reproducible biomarker discovery. *NeuroImage*, *223*(September). https://doi.org/10.1016/j.neuroimage.2020.117322

Vos de Wael, R., Benkarim, O., Paquola, C., Lariviere, S., Royer, J., Tavakol, S., Xu, T., Hong, S.-J., Langs, G., Valk, S., Misic, B., Milham, M., Margulies, D., Smallwood, J., & Bernhardt, B. C. (2020). BrainSpace: A toolbox for the analysis of macroscale gradients in neuroimaging and connectomics datasets. *Communications Biology*, *3*, 103. https://doi.org/10.1038/s42003-020-0794-7
